# A Discriminant Function Approach to Adjust for Processing and Measurement Error When a Biomarker is Assayed in Pooled Samples

**DOI:** 10.3390/ijerph121114723

**Published:** 2015-11-18

**Authors:** Robert H. Lyles, Dane Van Domelen, Emily M. Mitchell, Enrique F. Schisterman

**Affiliations:** 1 Department of Biostatistics and Bioinformatics, The Rollins School of Public Health of Emory University, 1518 Clifton Rd. N.E., Mailstop 1518-002-3AA, Atlanta, GA 30322, USA; E-Mail: dvandom@emory.edu; 2 Epidemiology Branch, Division of Intramural Population Research, Eunice Kennedy Shriver National Institute of Child Health and Human Development, Bethesda, MD 20892, USA; E-Mails: emily.mitchell@nih.gov (E.M.M.); schistee@mail.nih.gov (E.F.S.)

**Keywords:** epidemiology, errors-in-variables, odds ratio, pooling

## Abstract

Pooling biological specimens prior to performing expensive laboratory assays has been shown to be a cost effective approach for estimating parameters of interest. In addition to requiring specialized statistical techniques, however, the pooling of samples can introduce assay errors due to processing, possibly in addition to measurement error that may be present when the assay is applied to individual samples. Failure to account for these sources of error can result in biased parameter estimates and ultimately faulty inference. Prior research addressing biomarker mean and variance estimation advocates hybrid designs consisting of individual as well as pooled samples to account for measurement and processing (or pooling) error. We consider adapting this approach to the problem of estimating a covariate-adjusted odds ratio (OR) relating a binary outcome to a continuous exposure or biomarker level assessed in pools. In particular, we explore the applicability of a discriminant function-based analysis that assumes normal residual, processing, and measurement errors. A potential advantage of this method is that maximum likelihood estimation of the desired adjusted log OR is straightforward and computationally convenient. Moreover, in the absence of measurement and processing error, the method yields an efficient unbiased estimator for the parameter of interest assuming normal residual errors. We illustrate the approach using real data from an ancillary study of the Collaborative Perinatal Project, and we use simulations to demonstrate the ability of the proposed estimators to alleviate bias due to measurement and processing error.

## 1. Introduction

Epidemiological studies in general and environmental health-oriented research in particular often require conducting expensive laboratory assays of biospecimens to measure continuous biomarker concentrations that may be associated with adverse outcomes. In such studies, the physical pooling of samples prior to performing assays can be an effective design strategy aimed at reducing lab assay costs, ensuring or preserving sufficient specimen volumes, or minimizing the potential for measurements below a limit of detection. Specialized statistical techniques are required in order to extract the pertinent information from pooled specimens in a valid and efficient manner [1,2,3,4,5,6,7].

To effectively promote the concept of pooling in epidemiology, it is necessary to consider its application in the context of regression analysis (e.g., [8,9,10,11,12,13,14,15]). In this direction, Weinberg and Umbach [8] considered logistic regression with the goal of associating continuous levels of an exposure variable (measured in pools) with a binary outcome, adjusting for covariates. In discussing practical aspects of the approach, they anticipated the potential need to consider assay measurement errors in studies that utilize pooling. Along these lines, Schisterman *et al.* [7] proposed a framework for modeling measurement error as a feature of individual and pooled assay results, as well as pool processing error. The latter was assumed to apply only to assays made on pooled samples, resulting from the physical manipulations of individual specimens required to allocate them to pools. The prior considerations in [7] were limited to settings in which the objective is to estimate the unadjusted mean and variance of a biomarker assessed in pools.

In this paper, we focus on estimating the adjusted log odds ratio (log OR) relating a continuous exposure variable (X, measured in pools) to a binary outcome (Y), adjusting for covariates (**C**). As in prior related work, we assume each member of a given pool contributes the same sample aliquot volume and the lab assay is expected to return the arithmetic mean (equivalently, the sum) of biomarker concentrations across members of each pool [8,13]. The complication is that we seek to formally account for the fact that measurement error, processing error, or both may be incurred when applying the lab assay to measure X. Our approach relies upon discriminant function analysis (e.g., [16,17]), together with a prior paradigm for modeling sources of error [7]. We note that there is precedent for adopting the discriminant function approach to covariate measurement error problems [18,19]; however, this is to our knowledge the first attempt to apply it to analyses involving bioassay data obtained on pools. Our specific strategy utilizes a variant on classical discriminant function analysis, in which one assumes normal errors in a multiple linear regression model as opposed to multivariate normality of the exposure variable and any covariates [20].

Section 2 details the proposed methods, which are then applied in Section 3 to a motivating dataset stemming from a substudy of the Collaborative Perinatal Project [21,22] where the goal is to estimate the covariate-adjusted association between cytokine levels and the risk of spontaneous abortion. We study empirical properties of the proposed estimators using simulated data in Section 4, and discuss implications and future work in Section 5.

## 2. Methods

### 2.1. Models for Individual-Level Data without Measurement or Processing Error

Initially, assume the standard scenario in which individual-level data are available for a binary outcome (Y), a continuous exposure of interest (X), and for a set of covariates (**C**). The parameter of primary interest is the adjusted exposure log odds ratio (OR), commonly captured by the coefficient β in the following standard logistic regression model (Equation (1)):

(1) $$\text{logit}({\text{p}}_{\text{ij}})=\text{α}+\text{β}{\text{x}}_{\text{ij}}+\sum_{\text{t}=1}^{\text{T}}{\text{γ}}_{\text{t}}{\text{c}}_{\text{ijt}}$$

(i = 1,…, k; j = 1,…, g_i_; t = 1,…, T). Here p_ij_ = Pr (Y_ij_ = 1) where Y_ij_ is the binary outcome for the jth member of the ith of k eventual pools, and g_i_ is the number of specimens included in the ith pool (we discuss pooling further in the next section).

Prior to the widespread use of logistic regression, a discriminant function approach provided a convenient method to estimate the desired adjusted log OR (e.g., [16,17]). While this method has historically been less widely applied due to a requirement for additional distributional assumptions on (X, **C | Y**), Lyles, Guo and Hill [20] recently revisited this approach and demonstrated that the adjusted exposure log OR of interest can be efficiently estimated. In addition, their adaptation of the discriminant function approach required only a univariate distributional assumption for the errors in the following standard multiple linear regression (MLR) model (Equation (2)):

(2) $${\text{X}}_{\text{ij}}=\text{α}*+\text{β}*{\text{y}}_{\text{ij}}+\sum_{\text{t}=1}^{\text{T}}{\text{γ}}_{\text{t}}^{*}{\text{c}}_{\text{ijt}}+{\text{ε}}_{\text{ij}}$$

In particular, assuming that ${\text{ε}}_{\text{ij}}\overset{\text{iid}}{\sim }\text{N}(0,{\text{σ}}^{2})$, the same adjusted log OR targeted by β in Equation (1) is defined as the ratio β*/σ^2^ under Equation (2). When normality of the errors in Equation (2) holds, a uniformly minimum variance (UMVU) estimator for the log OR is available [20]. Further, simulation results [20] demonstrated that such a discriminant function-based estimator can be more accurate and precise in small samples than the standard maximum likelihood estimator (MLE) based on Equation (1).

### 2.2. Models for Pooled Data without Measurement or Processing Error

The first methodology designed to estimate the adjusted OR of interest when the exposure (X) is measured in pooled samples was introduced by Weinberg and Umbach [8]. They showed that if pools are homogeneous with respect to Y (*i.e.*, all members of pool i have y_ij_ = 0 or y_ij_ = 1), and if pooling is random within y strata, then model (1) implies the following poolwise logistic model (Equation (3)):

(3) $$\text{logit}({\text{p}}_{\text{i}})={\text{g}}_{\text{i}}\text{α}*+\text{β}{\text{x}}_{\text{i}}+\sum_{\text{t}=1}^{\text{T}}{\text{γ}}_{\text{t}}{\text{c}}_{\text{it}}+\text{ln}({\text{r}}_{\text{gi}})$$

where Y_i_ = 1 or 0 for a “case” or “control” pool (all members positive or negative, respectively), p_i_ = Pr (Y_i_ = 1 | X_i_ = x_i_, C_i1_ = c_i1_,…, C_iT_ = c_iT_ ), g_i_ is the size of (*i.e.*, number of specimens in) the i-th pool, ${\text{x}}_{\text{i}}=\sum_{\text{j}=1}^{{\text{g}}_{\text{i}}}{\text{x}}_{\text{ij}}$ is g_i_ times the average exposure across pool members assumed returned by the assay, ${\text{c}}_{\text{it}}=\sum_{\text{j}=1}^{{\text{g}}_{\text{i}}}{\text{c}}_{\text{ijt}}$ is the sum of the values of the t-th covariate across the members of pool i, and ln(r_gi_) is an offset with r_gi_ being the ratio of the number of case pools of size g_i_ to the number of control pools of size g_i_. Model (3) is fit with g_i_ as a covariate and with no intercept, applying the offset. This can be done using standard software, e.g., the LOGISTIC procedure in SAS [23], to obtain an MLE for β and its corresponding standard error.

While the Weinberg-Umbach approach provides a convenient method to estimate the log OR for a pooled exposure, it is not directly generalizable to incorporate information on pooling or measurement error. Thus, in the empirical studies that follow, our primary focus is on poolwise extensions of the model in Equation (2). Specifically, the MLR model for the summed exposure variable (X_i_) stemming from model (2) is:

(4) $${\text{X}}_{\text{i}}={\text{g}}_{\text{i}}\text{α}*+\text{β}*{\text{y}}_{\text{i}}^{*}+\sum_{\text{t}=1}^{\text{T}}{\text{γ}}_{\text{t}}^{*}{\text{c}}_{\text{it}}+{\text{ε}}_{\text{i}}$$

where X_i_ is a random variable corresponding to the observed sum (x_i_) of exposure levels across pool members, x_i_ and c_it_ are the same exposure and covariate sums that appear in Equation (3), ${\text{y}}_{\text{i}}^{*}=\sum_{\text{j}=1}^{{\text{g}}_{\text{i}}}{\text{y}}_{\text{ij}}$, and ${\text{ε}}_{\text{i}}=\sum_{\text{j}=1}^{{\text{g}}_{\text{i}}}{\text{ε}}_{\text{ij}}\overset{\text{iid}}{\sim }\text{N}(0,{\text{g}}_{\text{i}}{\text{σ}}^{2})$. Note that model (4), like model (3), is fit without an intercept and with the pool size (g_i_) as a covariate. If pool sizes are not equal, model (4) must be fit using weighted least squares (WLS) with weights w_i_ = 1/g_i_. This yields the WLS estimate $\hat{\text{β}}*$, along with the residual variance estimate ${\hat{\text{σ}}}^{2}=\text{MSE}={\left(\text{k}-\text{T}-2\right)}^{-1}\sum_{\text{i}=1}^{\text{k}}{\text{g}}_{\text{i}}^{-1}{\left({\text{Y}}_{\text{i}}-{\hat{\text{Y}}}_{\text{i}}\right)}^{2}$. Lyles, Guo and Hill [20] considered only standard MLR models (with an intercept) fit via ordinary least squares, but the distributional properties of $\hat{\text{β}}*$ and MSE assuming i.i.d. normal errors in the individual-level model (2) yield an immediate extension of their results to derive two estimators for the adjusted log OR of interest based on the WLS fit of model (4). To remain consistent with their notation, we refer to these as $\text{ln}{\left(\hat{\text{OR}}\right)}_{\text{samp}}$ and $\text{ln}{\left(\hat{\text{OR}}\right)}_{\text{umvu}}$, respectively, where “samp” denotes an unadjusted sample-based estimate. These new estimators for the adjusted log OR based on pooled exposure assays are given in (Equation (5)):

(5) $$\text{ln}{\left(\hat{\text{OR}}\right)}_{\text{samp}}=\hat{\text{β}}*/\text{MSE}\text{ and }\text{ln}{\left(\hat{\text{OR}}\right)}_{\text{umvu}}=\left(\frac{\text{k}-\text{T}-4}{\text{k}-\text{T}-2}\right)\text{ln}{\left(\hat{\text{OR}}\right)}_{\text{samp}}$$

Estimated variances also take the same form as those given in [20], *i.e.*, (Equation (6)):

(6) $$\text{V}\hat{\text{a}}\text{r}[\text{ln}{\left(\hat{\text{OR}}\right)}_{\text{samp}}]=\left(\frac{\text{k}-\text{T}-2}{\text{k}-\text{T}-4}\right)\text{MS}{\text{E}}^{-2}\times [\text{V}\hat{\text{a}}\text{r}(\hat{\text{β}}*)+2\hat{\text{β}}*/(\text{k}-\text{T}-2)].$$

As the subscript implies, $\text{ln}{\left(\hat{\text{OR}}\right)}_{\text{umvu}}$ is a minimum variance unbiased estimator of the adjusted log OR that accounts for pooling to assess exposure (X). This provides an alternative to the MLE of β based on the Weinberg-Umbach poolwise logistic model in Equation (3), if one is comfortable with the assumption of normal errors in model (4). In Section 4, we use simulation studies to investigate some of the statistical properties of these estimators.

### 2.3. Models for Pooled Data with Measurement and/or Processing Error

Schisterman *et al.* [7] proposed a framework for modeling measurement and processing errors when estimating the mean and variance of a continuous biomarker concentration based on pooled samples. We adapt their framework to address such errors when estimating the adjusted log OR of interest by augmenting the poolwise model in (4) for discriminant function analysis as follows (Equation (7)):

(7) $${\tilde{\text{X}}}_{\text{i}}={\text{g}}_{\text{i}}\text{α}*+\text{β}*{\text{y}}_{\text{i}}^{*}+\sum_{\text{t}=1}^{\text{T}}{\text{γ}}_{\text{t}}^{*}{\text{c}}_{\text{it}}+{\text{ε}}_{\text{i}}+{\text{ε}}_{\text{i}}^{\text{m}}+{\text{ε}}_{\text{i}}^{\text{p}}\times \text{I}({\text{g}}_{\text{i}}>1)$$

In model (7), the tilde (“~”) indicates that one observes an error-prone version of the desired sum of the exposures across members of pool i, while the new terms ${\text{ε}}_{\text{i}}^{\text{m}}$ and ${\text{ε}}_{\text{i}}^{\text{p}}$ represent measurement and processing errors, respectively. Following [7], we assume these errors are mutually independent with ${\text{ε}}_{\text{i}}^{\text{m}}\overset{\text{iid}}{\sim }\text{N}(0,{\text{σ}}_{\text{m}}^{2})$ and ${\text{ε}}_{\text{i}}^{\text{p}}\overset{\text{iid}}{\sim }\text{N}(0,{\text{σ}}_{\text{p}}^{2})$; we further assume these to be independent of the residual errors (ε_i_’s). Note that model (7) implies the assumptions that each laboratory assay result is subject to measurement error with a constant variance regardless of whether it is performed on a pooled or individual specimen, and the indicator function makes clear that each pooled assay (*i.e.*, where g_i_ > 1) is assumed subject to processing error with a constant variance regardless of the pool size.

Although iteratively reweighted least squares (IRWLS) could be an option, we take an ML approach to analysis based on model (7). Specifying the likelihood is straightforward, because the k poolwise outcomes ${\tilde{\text{X}}}_{\text{i}}$ are mutually independent such that ${\tilde{\text{X}}}_{\text{i}}|{\text{Y}}_{\text{i}}^{*},{\text{C}}_{\text{i}1},\ldots ,{\text{C}}_{\text{iT}}\sim \text{N}({\text{μ}}_{\text{i}},{\text{σ}}_{\text{i}}^{2})$, where (Equation (8)):

(8) $${\text{μ}}_{\text{i}}={\text{g}}_{\text{i}}\text{α}*+\text{β}*{\text{y}}_{\text{i}}^{*}+\sum_{\text{t}=1}^{\text{T}}{\text{γ}}_{\text{t}}^{*}{\text{c}}_{\text{it}}\text{ and }{\text{σ}}_{\text{i}}^{2}={\text{g}}_{\text{i}}{\text{σ}}^{2}+{\text{σ}}_{\text{m}}^{2}+{\text{σ}}_{\text{p}}^{2}\times \text{I}({\text{g}}_{\text{i}}>1)$$

The discriminant function-based MLE for the adjusted log OR then follows as the ratio of the MLEs of the respective parameters in (8), *i.e.*, (Equation (9))

(9) $$\text{ln}{\left(\hat{\text{OR}}\right)}_{\text{ml}}=\hat{\text{β}}*/{\hat{\text{σ}}}^{2},$$

with its delta method-based estimated variance given by (Equation (10)):

(10) $$\text{V}\hat{\text{a}}\text{r}[\text{ln}{\left(\hat{\text{OR}}\right)}_{\text{ml}}]=\text{V}\hat{\text{a}}\text{r}(\hat{\text{β}}*)/{\hat{\text{σ}}}^{4}+{\left(\hat{\text{β}}*/{\hat{\text{σ}}}^{4}\right)}^{2}\text{V}\hat{\text{a}}\text{r}({\hat{\text{σ}}}^{2}).$$

For computations, we use built-in SAS IML functions (NLPQN and NLPFDD) for numeric quasi-newton optimization and to approximate the observed information matrix [24].

### 2.4. Design Considerations and Bias Adjustment

From Equation (8), it is clear that the proposed discriminant function estimator in Equation (9) can only be used when the study design permits identifying the three separate variance components $\left({\text{σ}}^{2},{\text{σ}}_{\text{m}}^{2},{\text{σ}}_{\text{p}}^{2}\right)$, or at least under the condition that the residual variance (σ^2^) can be estimated uniquely. For this purpose, we recommend a “hybrid” design [7], in which individual exposure assay measurements (g_i_ = 1) are combined with pools (g_i_ > 1) of at least two different sizes. The pools of two or more sizes should permit estimation of σ^2^, while the individual assays provide observations devoid of processing error and should permit identification of the other two components. This requirement can be relaxed if one expects only measurement or processing error (not both). In that case one variance component $\left({\text{σ}}_{\text{p}}^{2}\text{ or }{\text{σ}}_{\text{m}}^{2}\right)$ is eliminated when specifying ${\text{σ}}_{\text{i}}^{2}$ in Equation (8), and a design featuring pools of any two or more sizes (including ‘pools’ of size 1) would theoretically be adequate. We return to such considerations in Section 3 when introducing the real data example, and we include “measurement error only” and “processing error only” models in simulation studies described in Section 4.

Given a design that allows estimation of a unique σ^2^, the stability of the discriminant function-based estimator in Equation (9) may remain an issue. We note that in the absence of measurement and processing error, the UMVU estimator in Section 2.2 improves stability by eliminating small-sample bias entirely. While we have not developed a UMVU estimator in the presence of measurement and/or processing error, a second-order Taylor series expansion leads to the following bias-adjusted alternative to the MLE in Equation (9):

(11) $$\text{ln}{\left(\hat{\text{OR}}\right)}_{\text{adj}}=\text{ln}{\left(\hat{\text{OR}}\right)}_{\text{ml}}-\hat{\text{β}}*\text{V}\hat{\text{a}}\text{r}({\hat{\text{σ}}}^{2})/{\hat{\text{σ}}}^{6}.$$

We would not recommend the use of Equation (11), for example, if the directionality of the log OR estimate changes relative to $\text{ln}{\left(\hat{\text{OR}}\right)}_{\text{ml}}$. Otherwise, Equation (11) acts as a shrinkage estimator that tends to have lower variability. While one could defend using the unadjusted standard error based on Equation (10) in conjunction with the bias-adjusted estimate (e.g., [25]), one could also contemplate multiplying that standard error by the ratio $\text{ln}{\left(\hat{\text{OR}}\right)}_{\text{adj}}/\text{ln}{\left(\hat{\text{OR}}\right)}_{\text{ml}}$. Multiplying by this ratio has no effect asymptotically, but may better reflect the variability of the adjusted estimator in small samples assuming that the ratio is approximately constant.

Clearly, one issue is that rare point estimates of σ^2^ close to 0 can correspond to exceedingly large log OR estimates. This instability makes the theoretical bias associated with both Equation (9) and Equation (11) infinite whenever there is a positive probability that ${\hat{\text{σ}}}^{2}$ equals 0, and can also produce occasional “blow ups” in estimated standard errors based on Equation (10). For this reason, our empirical studies in Section 4 include a discussion of practical strategies to reduce such problems. This includes consideration of Akaike’s information criterion (AIC; [26]) to select a model accounting solely for measurement or processing error if the model accounting for both is subject to instability in the estimated log OR and/or its accompanying standard error.

## 3. Example

### 3.1. Collaborative Perinatal Project Data

The Collaborative Perinatal Project (CPP) was originally conducted during the years 1959–1974 to study exposures and outcomes related to pregnancy [21]. In a subsequent nested case-control study using stored serum, investigators assayed cytokine concentrations in controls and in cases who experienced spontaneous abortion (SA) [22]. As part of this substudy, cytokines were measured in individual samples as well as in pools of size 2. We analyze data from 666 women, for whom the variables SA status (Y; 0 or 1), race (C_1_; black *vs*. white), and smoking status (C_2_; yes *vs*. no) were measured individually. The cytokine concentration (X) of interest is that of monocyte chemotactic protein 1 (MCP1; X). We use MCP1 assay results from 251 pools of size 2 (involving 502 women), along with individual MCP1 assays from the other 164 women who were not included in pools. Women paired in pools were matched on SA (Y) status.

Basic descriptive statistics for the 666 participants include the following: 305 (46%) had SA = 1 (including 85 individuals and 110 pairs whose serum was pooled), 189 (28%) were black, and 313 (47%) smoked. We consider four models based on Equation (7), distinguished by the extent to which they account for sources of error: (a) both measurement error (ME) and pool processing error (PE); (b) ME only; (c) PE only; (d) neither ME nor PE. The parameter of interest is the log OR associating individual-level SA status (Y) with a unit increase in MCP1 concentration (X), adjusting for race (C_1_) and smoking status (C_2_).

### 3.2. Results

Table 1 summarizes analyses carried out for the CPP ancillary study. The first thing to note is that when both measurement and processing error are accounted for in model (8), the parameter β* is identifiable, but the residual variance (σ^2^) and hence the log OR of interest is not. This stems from the fact that only a single pool size (k_i_ = 2) was utilized in the study (see Section 2.4). The other three models (ME only, PE only, and neither ME nor PE) all agree with regard to a positive but non-significant estimated log OR characterizing the adjusted association between SA status and MCP1 levels. For the ME only model, the estimated measurement error variance $\left({\text{σ}}_{\text{m}}^{2}\right)$ attained a lower bound (0.001) that was set for each variance component in the numerical ML optimization process. As such, results for the ME only and the “neither ME nor PE” models are extremely similar to each other. Those results are also qualitatively similar to an analysis based on the Weinberg-Umbach [8] model in Equation (3), results of which we provide in the last row for comparison.

While the analysis suffers from lack of identifiability with respect to the most general model, note that AIC strongly favors the PE only model over the other two viable choices. The MLE for the log (OR) is also noticeably higher (0.39 *vs*. 0.31) under this model, although the accompanying standard error is also larger and the inferential result remains non-significant. As a final note, Table 1 illustrates that the proposed bias-corrected log OR estimates and adjusted standard errors (Section 2.4) are very similar to the uncorrected MLEs for this example, given the relatively small effect and large sample size.

**Table 1:** Analysis of CPP Substudy Data Including 164 Individual and 251 Pooled MCP1 Assays ^a^.

Model	$\hat{\text{β}}*$	${\hat{\text{σ}}}^{2}$	${\hat{\text{σ}}}_{\text{m}}^{2}$	${\hat{\text{σ}}}_{\text{p}}^{2}$	$\text{ln}{\left(\hat{\text{OR}}\right)}_{\text{ml}}$	$\text{ln}{\left(\hat{\text{OR}}\right)}_{\text{adj}}$ ^e^	AIC
ME and PE ^b^	0.031 (0.026)	--	--	--	--	--	--
ME only	0.032 (0.025)	0.102	0.001 ^c^	--	0.311 (0.25) [−0.17, 0.80]	0.310 (0.25) [−0.17, 0.79]	420.64
PE only	0.031 (0.026)	0.079	--	0.078	0.388 (0.32) [−0.25, 1.02]	0.383 (0.32) [−0.25, 1.01]	412.82
Neither ME nor PE	0.032 (0.025)	0.103	--	--	0.309 (0.25) [−0.17, 0.79]	0.308 (0.24) [−0.17, 0.79]	418.46
Logistic regression ^d^	--	--	--	--	0.270 (0.24) [−0.20, 0.74]	--	--

^a^ Numbers in parentheses () are estimated standard errors; 95% CIs are in brackets []; ^b^ Model fails to identify σ^2^ due to design limitations (k_i_ = 1,2 only); ^c^ ${\hat{\text{σ}}}_{\text{m}}^{2}$ hits boundary constraint of 0.001; ^d^ Based on Weinberg-Umbach poolwise model (Section 2.2), not accounting for ME or PE; ^e^ Estimates and standard errors adjusted as proposed in Section 2.4.

## 4. Simulations

We conducted simulations to study empirical properties of the estimators discussed in Section 2, with data generated to closely mimic the CPP ancillary study. Specifically, a Bernoulli variable C_2_ was first generated to match the observed prevalence of smoking status. A second Bernoulli variable C_1_ was then drawn with prevalence matching that observed within the corresponding smoking group. Then, SA status (Y) was generated conditional on C_1_ and C_2_, according to a logistic regression with parameters matching estimates obtained from the observed CPP data. Finally, MCP1 concentration (X) was generated according to model (2), again with parameters closely mimicking estimates obtained from the corresponding model fit to individual-level CPP data. We note that this means of generating the data can also be shown to imply the validity of model (1). The true values of β* and σ^2^ used in the simulations were 0.035 and 0.08, respectively, for a true adjusted log OR of 0.4375.

Unlike the actual CPP substudy which involved only one pool size together with individual samples (k_i_ = 1,2), we generated data with three pool sizes (k_i_ = 1,2,3) so that all variance components in Equation (8) would theoretically be identifiable. Specifically, 50% of the total number (N) of individual “subjects” were allocated to pools of size 4, 50% of the remainder to pools of size 2, and the rest were treated individually. True poolwise exposures (X_i_) were calculated as the sum of individual exposures for those in the pool. For simulations involving ME and/or PE, normal errors were randomly generated with mean 0 and variance ${\text{σ}}_{\text{m}}^{2}=0.08$ and/or ${\text{σ}}_{\text{p}}^{2}=0.08$, respectively.

### 4.1. Results of Simulations with Neither ME nor PE

We conducted initial simulations to study empirical properties of the estimators discussed in Section 2.2, for use in the absence of measurement and pool processing error. Two overall sample sizes (N = 2000 and N = 200) were considered. Table 2 demonstrates the anticipated unbiasedness of the WLS estimators $\hat{\text{β}}*$ and ${\hat{\text{σ}}}^{2}$ under either sample size, along with virtual unbiasedness empirically of the estimators $\text{ln}{\left(\hat{\text{OR}}\right)}_{\text{samp}}$ and $\text{ln}{\left(\hat{\text{OR}}\right)}_{\text{umvu}}$ when N = 2000. With this sample size, the Weinberg-Umbach model [8] also yields a log OR estimator with very similar properties.

For a small overall sample size (N = 200) we note that both $\text{ln}{\left(\hat{\text{OR}}\right)}_{\text{samp}}$ and $\text{ln}{\left(\hat{\text{OR}}\right)}_{\text{umvu}}$ remain highly stable, while the theoretical unbiasedness and efficiency advantage associated with $\text{ln}{\left(\hat{\text{OR}}\right)}_{\text{umvu}}$ begins to show empirically. In this case, the logistic regression-based estimator suffers in terms of bias and precision relative to the discriminant function-based log OR estimators that take advantage of the assumption of normal residual errors. Similar findings in the standard setting without pooling were discussed by Lyles, Guo and Hill [20].

**Table 2:** Simulations Under Model with Neither ME nor PE to Assess Estimators in Section 2.2 ^a,b^.

N	$\hat{\text{β}}*$	MSE	$\text{ln}{\left(\hat{\text{OR}}\right)}_{\text{samp}}$	$\text{ln}{\left(\hat{\text{OR}}\right)}_{\text{umvu}}$	Logistic Regression ^c^
2000	0.035 (0.013)	0.080	0.439 (0.166) [94.6%]	0.438 (0.166) [94.6%]	0.441 (0.168) [94.8%]
200	0.035 (0.042)	0.080	0.447 (0.545) [95.8%]	0.438 (0.534) [95.8%]	0.474 (0.586) [95.1%]

^a^ Table shows mean estimates across 5000 simulations, with empirical standard deviations in parentheses () and 95% CI coverages in brackets []; ^b^ True values: β* = 0.035, σ^2^ = 0.080, ln(OR) = 0.438; ^c^ Based on Weinberg-Umbach poolwise model.

### 4.2. Results of Simulations with ME and/or PE

We considered three overall sample sizes (N = 2000, 1000, 500) in simulations accounting for measurement and/or processing error. While the inclusion of three pool sizes (k_i_ = 1,2,3) permits identifying all variance components in the general model that requires estimating both ${\text{σ}}_{\text{m}}^{2}$ and ${\text{σ}}_{\text{p}}^{2}$ in addition to the residual variance σ^2^, some numerical instabilities were still observed. Specifically, the MLE for σ^2^ occasionally met the lower boundary of 0.001, and/or the estimated standard error accompanying $\text{ln}{\left(\hat{\text{OR}}\right)}_{\text{ml}}$ in Equation (9) was implausibly large. In such boundary cases or if the estimated standard error under the general model was more than 10 times the standard error under a model ignoring ME and PE, we used the AIC criterion to select the best fitting alternative model (ME only or PE only) to estimate the log OR. Such model selection adjustments were fairly common with N = 500 when generating data under the most general model, but were almost never necessary under the ME only or PE only models and were much less frequent for larger sample sizes. If a different model than the one that generated the data was selected under the specified criteria, standard errors as well as the point estimate of the log OR were based on the selected model.

Table 3 provides results for data simulated under the general model with ${\text{σ}}^{2}={\text{σ}}_{\text{m}}^{2}=$ ${\text{σ}}_{\text{p}}^{2}=0.08$. We focus mainly on the summary for the MLE of the log OR, in which we include the mean and median across 2500 simulations to get a sense of both mean and median bias. As indicated in the footnotes, instability in the estimate of σ^2^ or in the standard error accompanying $\text{ln}{\left(\hat{\text{OR}}\right)}_{\text{ml}}$ led to an AIC-based decision to base estimation on the ME only or PE only model in 19.1% of simulations with N = 500. This percentage reduced to 8.5% and 3.4% with N = 1000 and 2000, respectively. Ultimately, the estimator is characterized by acceptable mean and median bias and accompanied by adequate (if a bit anticonservative) CI coverages. As expected, the Weinberg-Umbach model (Section 2.2) produces a markedly attenuated log OR estimate with sub-nominal coverage, since it does not account for measurement or processing errors. We do not summarize the bias-corrected estimator $\text{ln}{\left(\hat{\text{OR}}\right)}_{\text{adj}}$ in Table 3, since numerical issues affecting $\text{ln}{\left(\hat{\text{OR}}\right)}_{\text{ml}}$ also tended to impact stability of the estimated correction factor under the most general model.

Table 4 provides results for data simulated under the ME only model with ${\text{σ}}^{2}={\text{σ}}_{\text{m}}^{2}=$0.08. Mean log OR estimates were much closer to their medians than under the general model (Table 3), so we report mean estimated standard errors in place of median estimates. These match empirical SDs closely, and translate to reasonable CI coverages. Here we see the value of the bias-corrected estimator $\text{ln}{\left(\hat{\text{OR}}\right)}_{\text{adj}}$ in Equation (11), with respect to accuracy as well as precision (particularly for smaller overall sample sizes). In this case we continue to see marked bias in the logistic regression-based estimator that ignores measurement error.

**Table 3:** Simulations Under Model with Both ME and PE to Assess Estimators in Section 2.3 ^a,b,c^.

N	$\hat{\text{β}}*$	${\hat{\text{σ}}}^{2}$	${\hat{\text{σ}}}_{\text{m}}^{2}$	${\hat{\text{σ}}}_{\text{p}}^{2}$	$\text{ln}{\left(\hat{\text{OR}}\right)}_{\text{ml}}$ ^d^	Logistic Regression ^e^
2000	0.035 (0.017)	0.079	0.081	0.082	0.474 |0.438| (0.28) [95.4%]	0.254 |0.254| (0.13) [66.7%]
1000	0.035 (0.024)	0.077	0.082	0.081	0.463 |0.417| (0.37) [96.2%]	0.252 |0.251| (0.18) [79.4%]
500	0.035 (0.034)	0.077	0.081	0.080	0.448 |0.402| (0.49) [97.2%]	0.259 |0.254| (0.26) [88.9%]

^a^ Table shows mean estimates across 2500 simulations, with median estimates in bars ||, empirical standard deviations in parentheses () and 95% CI coverages in brackets []; ^b^ True values: β* = 0.035, ${\text{σ}}^{2}={\text{σ}}_{\text{m}}^{2}={\text{σ}}_{\text{p}}^{2}=0.08$, ln(OR) = 0.438; ^c^ Mean estimates of β* and variance components exclude simulation runs in which σ^2^ estimate hit 0.001 boundary. This occurred in 7.6%, 1.2%, and 0.08% of runs with N = 500, 1000, 2000, respectively; ^d^ Final log OR estimate incorporates AIC-based model selection (see Section 4.2) with ME only or PE only model selected in 19.1%, 8.5%, and 3.4% of runs with N = 500, 1000, 2000, respectively; ^e^ Based on Weinberg-Umbach poolwise model (Section 2.2), not accounting for ME or PE.

**Table 4:** Simulations Under ME Only Model to Assess Estimators in Section 2.3 ^a,b^.

N	$\hat{\text{β}}*$	${\hat{\text{σ}}}^{2}$	${\hat{\text{σ}}}_{\text{m}}^{2}$	$\text{ln}{\left(\hat{\text{OR}}\right)}_{\text{ml}}$	$\text{ln}{\left(\hat{\text{OR}}\right)}_{\text{adj}}$	Logistic regression ^d^
2000	0.035 (0.016)	0.079	0.080	0.448 (0.22) [0.21] {95.4%}	0.438 (0.21) [0.21] {95.6%}	0.291 (0.13) [0.13] {79.9%}
1000	0.035 (0.021)	0.079	0.080	0.474 (0.32) [0.31] {96.5%}	0.450 (0.30) [0.30] {96.4%}	0.298 (0.19) [0.19] {89.0%}
500	0.036 (0.031)	0.076	0.083	0.522 c (0.51) [0.50] {97.5%}	0.454 c (0.42) [0.43] {96.8%}	0.307 (0.28) [0.27] {92.0%}

^a^ Table shows mean estimates across 2500 simulations, with empirical standard deviations in parentheses (), mean estimated standard errors in brackets [] and 95% CI coverages in braces {}; ^b^ True values: β* = 0.035, ${\text{σ}}^{2}={\text{σ}}_{\text{m}}^{2}=0.08$, ln(OR) = 0.438; ^c^ Final log OR estimate incorporates AIC-based model selection (see Section 4.2) with PE only model selected in 0.5% of runs with N = 500. ME only model used in 100% of runs with N = 1000 and 2000; ^d^ Based on Weinberg-Umbach poolwise model (Section 2.2), not accounting for ME or PE.

Table 5 provides results for data simulated under the PE only model with ${\text{σ}}^{2}={\text{σ}}_{\text{p}}^{2}=$0.08. This model encountered no numerical difficulties at any of the three sample sizes, and in this case we see very little bias in either of the two discriminant function-based estimators. Attenuation remains present in the logistic regression-based estimator, but is less prominent given that processing error only impacts pooled (and not individual) simulated assay values.

**Table 5:** Simulations Under PE Only Model to Assess Estimators in Section 2.3 ^a,b,c^.

N	$\hat{\text{β}}*$	${\hat{\text{σ}}}^{2}$	${\hat{\text{σ}}}_{\text{p}}^{2}$	$\text{ln}{\left(\hat{\text{OR}}\right)}_{\text{ml}}$	$\text{ln}{\left(\hat{\text{OR}}\right)}_{\text{adj}}$	Logistic Regression
2000	0.035 (0.014)	0.080	0.080	0.444 (0.18) [0.18] {95.6%}	0.442 (0.18) [0.18] {95.5%}	0.356 (0.15) [0.15] {91.3%}
1000	0.035 (0.020)	0.079	0.078	0.441 (0.26) [0.26] {95.0%}	0.438 (0.26) [0.26] {95.0%}	0.356 (0.21) [0.21] {92.4%}
500	0.035 (0.029)	0.079	0.078	0.447 (0.37) [0.37] {96.0%}	0.440 c (0.37) [0.36] {96.0%}	0.361 (0.30) [0.30] {94.3%}

^a^ Table shows mean estimates across 2500 simulations, with empirical standard deviations in parentheses (), mean estimated standard errors in brackets [] and 95% CI coverages in braces {}; ^b^ True values: β* = 0.035, ${\text{σ}}^{2}={\text{σ}}_{\text{p}}^{2}=0.08$, ln(OR) = 0.438; ^c^ PE only model used in 100% of all runs for each sample size; ^d^ Based on Weinberg-Umbach (1999) poolwise model (Section 2.2), not accounting for ME or PE.

## 5. Discussion

Despite the potential cost benefits and facilitation of lab assay procedures afforded by pooling, possible measurement and processing errors associated with individual and/or pooled measurements may limit its use in practice. In proposing set-based logistic regression with a continuous exposure variable measured in pools, Weinberg and Umbach [8] anticipated and provided insights into the potential effects of measurement errors. However, to our knowledge, this report offers the first demonstration of a comprehensive and implementable approach to adjust for ME, PE, or both when the goal is to estimate an adjusted exposure odds ratio. We used a discriminant function-based strategy (e.g., [16,20]) to convert the problem to a multiple linear regression framework. For modeling measurement and processing errors in the context of pooled exposures, we then followed the paradigm proposed by Schisterman *et al.* [7]. Combining these two methodologies allows us to target the individual-level adjusted log OR associating exposure with a binary outcome in the presence of pooling, ME and PE.

Our method can be further extended to account for more sources of error (e.g., attributable to technician, variations in temperature, *etc.*), and to allow the processing error variance ${\text{σ}}_{\text{p}}^{2}$ to vary with pool size. However, this will require a more complex hybrid design, where different pool sizes are available as a function of the variance components. We also note in passing that inclusion of replicate assay results taken on individual specimens could offer an alternate means of identifying the measurement error variance, ${\text{σ}}_{\text{m}}^{2}$.

Mitchell *et al.* [14] provided ML methodology applicable to model (4) when the exposure variable X is right-skewed, as is common in environmental epidemiologic studies. We believe that ongoing research in the direction of extensions to permit adjustment for ME and/or PE in that scenario could be highly valuable. Further work could also focus more specifically on study design considerations (see Section 2.4), e.g., determining an optimal allocation of pool sizes. Such an effort may benefit from past work demonstrating efficiency gains possible in the context of model (4) when pool allocations are made with respect to other covariates (C) in addition to the outcome (Y) [15]. Nevertheless, we expect that pooling with respect to Y alone will typically be highly efficient when targeting the parameter β* in model (4), and thus also for the purpose of estimating the adjusted log OR of interest. It is worth noting that the discriminant function approach (with and without accounting for measurement or processing errors) readily accommodates pool allocations that are completely random (*i.e.*, not made within separate strata of Y or any other variable), as well as allocations that are dependent on Y in addition to other covariates. We caution that the former strategy is less statistically efficient than y-dependent pooling, which is a requirement for use of the Weinberg-Umbach poolwise logistic regression model [8]. Even ignoring ME and/or PE, the latter model requires offset adjustments if pooling is dependent on both Y and covariates [13,27].

Finally, we are currently exploring methods to adjust for ME and PE rooted in the logistic regression, as opposed to the discriminant function framework. Although the discriminant function method is much simpler computationally, an advantage of the logistic regression approach would be the ability to estimate adjusted ORs associated with other covariates in addition to the exposure OR. We anticipate building naturally off of considerations given here, in addition to prior related references [7,8,13].

The Appendix contains SAS/IML code that was used in fitting model (7) to the CPP example data. Only minor adjustments are required to alter this code to account for ME or PE only. Readers interested in implementing the approach described are encouraged to direct any remaining computational questions to the first author.

## 6. Conclusions

When a biomarker is assayed in pools, a discriminant function-based approach in combination with an existing framework for modeling measurement and processing errors proves feasible and useful when the goal is to account for such errors in regression modeling to estimate an adjusted exposure odds ratio.

## Appendix: SAS/IML Code Used to Fit Model (7) to Example Data

The following is computer code used to fit model (7) to the CPP ancillary study data, accounting for both ME and PE. This code is provided to assist with implementation of the ML analysis, even though (as discussed in Section 3.2) the variance components in the general ME/PE model were not identifiable due to inclusion of only one pool size > 1 in the study. The SAS dataset “fordiscrim” contained the following variables: ki (size of the ith pool; these took values of 1 and 2 in the example), SAsum (sum of indicators for SA status (Y) for each pool), smokesum (sum of indicators for smoking status (C_1_) for each pool), racesum (sum of indicators for ethnicity group (C_2_) for each pool), mcp1_sum (pooled (or individual, for ki = 1) MCP1 assay value (X) for each pool).

proc iml worksize = 70 symsize = 250;

use fordiscrim;
 read all var{ki} into kj;
 read all var{SAsum} into ystar;
 read all var{smokesum} into smokestar;
 read all var{racesum} into racestar;
 read all var{mcp1_sum} into xstrtilde;   
close fordiscrim;
npools = 415; ** 251 pools of size 2, and 164 individual samples **;

** Specifying likelihood for FULL ML method **;

START LIKELIC(parms) global (npools,kj,pi,ystar,smokestar,racestar,xstrtilde);

bet0prm = parms [1];
bet1prm = parms [2];
gamm1prm = parms [3]; *** Parameters in model (7) to be estimated ***;
gamm2prm = parms [4];
sigsqx = parms [5];
sigsqp = parms [6];
sigsqm = parms [7];

pi = 2 * arsin (1);

*** NOTE: LOWER BOUND CONSTRAINT ON VARIANCE COMPONENTS FOR 
STABILITY ***;

sigsqx = max (sigsqx,.001);
sigsqp = max (sigsqp,.001);
sigsqm = max (sigsqm,.001);

* contributions to likelihood ;

        func_lkC = j (npools,1,.);

        do u = 1 to npools;

                ystr = ystar [u,1];
                smkstr = smokestar [u,1];
                racestr = racestar [u,1];
                ki = kj [u,1];
                xstrt = xstrtilde [u,1];

 kigt1 = 0;
 if ki > 1 then kigt1 = 1;

muxtstrgyc = ki#bet0prm + bet1prm#ystr + gamm1prm#smkstr + gamm2prm#racestr;
sigsqxtstrgyc = ki#sigsqx + sigsqp#kigt1 + sigsqm;

  func_lkC[u,1] = (1/sqrt(2#pi#max(sigsqxtstrgyc,1E-4)))#exp(-(xstrt-muxtstrgyc)##2/(2#max(sigsqxtstrgyc,1E-4)));

** Next 2 lines to prevent instability during iterations **;

          if func_lkC [u,1] < 1E-100 then func_lkC [u,1] = 1E-100;
          if func_lkC [u,1] > 1E20 then func_lkC [u,1] = 1E20;

           func_lkchk = func_lkC [u,1];

           * print func_lkchk;

        end;

  m2loglikC = -2 # sum (log(func_lkC)) ;

   return (m2loglikC);

FINISH LIKELIC;

**********************************************************************
The following calls the minimization function, computes the Hessian, etc.
**********************************************************************;

START COMPC; ** Maximum likelihood method **;

   * create vector of initial parameter estimates for function;

   parms =.2||.2||.2||.2||.5||.5||.5;

   * options vector for minimization function;

   option = {0 3};

  ** matrix of lower (row 1) and upper (row 2) bound
   constraints on probabilities **;

   con={. . . . .001 .001 .001,        . . . . . . .};

   *call function minimizer in IML;

   call nlpqn(rc,xres, “likelic”,parms,option,con);

   * create vector of mles computed using function minimizer;

   Parms = xres`;

   * compute numerical value of Hessian( and covariance matrix)
   using mles calculated above ;

  print parms;

   * call function to approximate 2nd derivatives for Hessian;

  call NLPFDD (crit,grad,hess, “likelic”,parms);

  cov_mat = 2 * inv (hess);
  se_vec1 = sqrt (vecdiag (cov_mat));

   print se_vec1;
   print cov_mat;

   print rc;

bet0prm = parms [1];
bet1prm = parms [2];
gamm1prm = parms [3];
gamm2prm = parms [4];
sigsqx = parms [5];
sigsqp = parms [6];
sigsqm = parms [7];

sebet1prm = sqrt (cov_mat [2,2]);
segamm1prm = sqrt (cov_mat [3,3]);

bet1discrim = bet1prm/sigsqx;

print bet1discrim;

FINISH COMPC;

run compc;

QUIT;
